# Comparação entre Cinco Escores de Risco em Pacientes com Síndromes Coronárias Agudas Submetidos à Revascularização Cirúrgica durante a Internação Índice

**DOI:** 10.36660/abc.20250320

**Published:** 2025-10-08

**Authors:** José C. Nicolau, Roberto R. C. V. Giraldez, Fabio B. Jatene, Luís A. O. Dallan, Luiz A. Lisboa, Omar A. V. Mejia, Jorge L. M. Ribera, Adriadne J. Bertolin, Luciano M. Baracioli, Felipe G. Lima, Maria C. D. Andrade, Santiago A. C. Vintimilla, Leonardo Salis, Fabiane L. de Freitas, Maxim Goncharov, Lucas C. Godoy, Remo H. de M. Furtado, Michael E. Farkouh

**Affiliations:** 1 Hospital das Clínicas Faculdade de Medicina Universidade de São Paulo São Paulo SP Brasil Instituto do Coracao do Hospital das Clínicas da Faculdade de Medicina da Universidade de São Paulo, São Paulo, SP – Brasil; 2 University of Toronto Peter Munk Cardiac Centre Toronto Canadá University of Toronto – Peter Munk Cardiac Centre, Toronto – Canadá; 3 Cedars-Sinai Health System Los Angeles EUA Cedars-Sinai Health System, Los Angeles, Califórnia – EUA

**Keywords:** Síndrome Coronária Aguda, Mortalidade, Revascularização Miocárdica, Continuidade da Assistência ao Paciente

## Abstract

**Fundamento:**

Os escores de risco (ER) para pacientes com síndromes coronárias agudas (SCA) e cirurgia de revascularização do miocárdio (CRM) foram testados anteriormente, mas pouco se sabe sobre seu valor em pacientes com SCA submetidos à CRM durante a internação índice.

**Objetivo:**

Comparar cinco diferentes ER em pacientes com SCA submetidos à CRM durante a internação índice.

**Métodos:**

Os ER analisados foram GRACE, escore TIMI para SCA sem supradesnivelamento do segmento ST (TIMI-NSTEACS), escore TIMI para infarto agudo do miocárdio com supradesnivelamento do ST (TIMI-STEMI), escore de sangramento dos estudos ACCUITY/HORIZONS (A-H) e EuroSCORE II. Os ER foram avaliados quanto ao seu desempenho durante a fase intra-hospitalar e o acompanhamento a longo prazo após a alta. Considerou-se significativo um valor de p <0,05.

**Resultados:**

Um total de 999 pacientes foi incluído entre 1998 e 2022. O tempo médio entre o início dos sintomas e a CRM foi de 6,3 ± 5,5 dias. As áreas sob as curvas ROC foram 0,82 (IC 95% 0,74 – 0,89; p<0,001) para GRACE; 0,78 (IC 95% 0,62-0,93; p=0,004) para TIMI-STEMI; 0,75 (IC 95% 0,61-0,83; p<0,001) para EuroSCORE II; 0,67 (IC 95% 0,59-0,76; P<0,001) para sangramento A-H e 0,58 (IC 95% 0,49-0,67; p=0,131) para TIMI-NSTEACS. Excluindo-se os óbitos intra-hospitalares, apenas o GRACE e o TIMI-STEMI foram significativamente associados à mortalidade a longo prazo (seguimento médio de 5,5 ± 4 anos). Nas análises multivariadas, o escore GRACE foi o único ER significativamente associado à mortalidade intra-hospitalar e em longo prazo em todos os modelos ajustados.

**Conclusão:**

Em pacientes com SCA submetidos à CRM durante a internação-índice, o escore GRACE foi o único escore de risco que permaneceu independentemente associado à mortalidade intra-hospitalar e em longo prazo em todos os modelos desenvolvidos, após ajuste para potenciais fatores de confusão. Além disso, o escore GRACE teve melhor desempenho do que outros na predição de óbitos intra-hospitalares. Esses achados podem influenciar o processo de tomada de decisão clínica nessa população de alto risco.

## Graphical abstract

**Figura Central:**
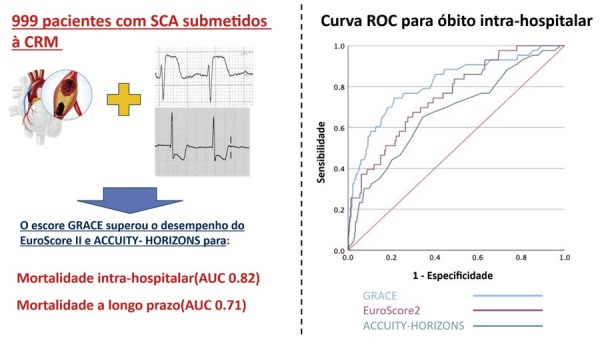
Comparação entre Cinco Escores de Risco em Pacientes com Síndromes Coronarianas Agudas Submetidos à Revascularização Cirúrgica durante a Internação Índice

## Introdução

As doenças cardiovasculares são a principal causa de morte em todo o mundo, sendo a doença isquêmica do coração a mais prevalente entre elas.^1^ Em pacientes com síndromes coronárias agudas (SCA), cerca de 40% apresentam doença arterial coronariana multiarterial.^2^ Apesar da diminuição das taxas de utilização da revascularização cirúrgica coronariana, principalmente relacionada ao uso generalizado da intervenção coronária percutânea (ICP), até 10%-15% desses pacientes são encaminhados para cirurgia de revascularização do miocárdio (CRM) durante a internação índice da SCA,^3 - 5^ com alguma evidência de benefício da CRM sobre a ICP nessa população, especialmente em pacientes com diabetes.^6 - 10^Nos EUA, aproximadamente 35% dos pacientes com diabetes e SCA sem supradesnivelamento do segmento ST (NSTEACS) são submetidos à CRM durante a internação índice.^11^

Os escores cardiovasculares em geral apresentam limitações.^12^ Diferentes escores foram desenvolvidos para a estratificação de risco em pacientes com SCA, avaliando sangramento (escore de sangramento ACUITY/HORIZONS)^13^ ou eventos isquêmicos (escore GRACE),^14^ em pacientes com infarto do miocárdio com supradesnivelamento do segmento ST (escore TIMI-STEMI)^15^ ou com NSTEACS (TIMI-NSTEACS score).^16^ Além disso, escores de risco cardíaco para predizer mortalidade pós-cirurgia cardíaca de grande porte, como o EuroSCORE II, foram validados.^17^ Curiosamente, uma maior proporção de utilização de ICP, em comparação com a CRM (maior taxa de ICP: CRM), parece estar associada a um risco aumentado de mortalidade hospitalar associada à CRM entre pacientes com SCA.^5^

Até onde sabemos, esses escores de risco não foram testados em uma população não selecionada do mundo real de pacientes com SCA submetidos à CRM durante a hospitalização índice. Este estudo tem como objetivo comparar o desempenho de vários escores de risco em pacientes com SCA submetidos à CRM durante a internação índice, com foco na mortalidade por todas as causas intra-hospitalar. A principal análise secundária foi a comparação entre os escores em longo prazo após a alta na população com dados disponíveis.

## Materiais e métodos

### Conformidade com os padrões éticos

Este estudo está em conformidade com as normas do *International Council for Harmonization* (ICH) sobre pesquisa médica em humanos. O estudo foi aprovado pelo Comitê de Ética do Hospital das Clínicas. Como os dados são baseados em informações individuais obtidas para fins administrativos, o consentimento informado foi dispensado, de acordo com a regulamentação local.

### População

A partir de um banco de dados administrativo coletado prospectivamente no Instituto do Coração da Universidade de São Paulo, um centro médico terciário acadêmico, analisamos retrospectivamente 999 pacientes consecutivos admitidos com SCA na Unidade Coronária (mediana de idade de 64 anos, 70,2% do sexo masculino) que foram submetidos à CRM durante a internação-índice de 1999 a 2022. A Figura suplementar 1 resume as inclusões ao longo desse período. O tempo médio entre o início dos sintomas e a revascularização do miocárdio foi de 6,3 ± 5,5 dias. Todos os pacientes estavam em uso de dupla antiagregação plaquetária. Com exceção de 95 pacientes participantes de um estudo randomizado em que a estratégia de tempo para envio do paciente para CRM foi baseada na avaliação da função plaquetária,^18^ o tempo de espera para a descontinuação do P2Y12 e CRM foi de 5 a 7 dias, seguindo a recomendação das diretrizes.^19^ Os pacientes com IAMCSST foram frequentemente tratados inicialmente com terapia fibrinolítica nas instituições de referência antes de serem transferidos para o nosso centro (tempo médio de 1,04 ± 1,83 dias). Os dados coletados incluíram variáveis demográficas, bem como características de apresentação do evento índice. Em relação ao acompanhamento de longo prazo, apesar de nosso banco de dados ter sido congelado em 2022, a última tentativa de contato com todos os pacientes foi realizada em 2015 (o tempo máximo de acompanhamento foi de 16 anos, com média de 5,52 anos), e 249 pacientes foram incluídos entre 2015 e 2022, correspondendo a 24,9% dos pacientes perdidos no seguimento. Ao final, obtivemos o status de sobrevida de 597 pacientes para toda a população (59,76%) e 314 pacientes para a população com dados de escore completo (62,3%).

### Escores

Todos os escores analisados são amplamente utilizados e já foram validados em várias publicações.^16 , 17 , 20^ Dado o período de coleta de dados, alguns dos dados dos escores foram coletados retrospectivamente, sendo os dados do EuroSCORE-II^17^ e outras variáveis cirúrgicas coletadas inteiramente de maneira retrospectiva dos prontuários dos pacientes e de outras fontes hospitalares por dois dos autores (FLF, OAVM) e adicionados ao banco de dados original. Em relação ao escore de sangramento A-H, dado o fato de a bivalirudina não estar disponível no país, este item foi sempre computado como zero no escore. Para o escore GRACE, foi utilizada a versão 2.0.^20^ O escore foi calculado com base nas informações coletadas em nosso banco de dados e que são necessárias para o cálculo do escore (idade, creatinina, desvio do segmento ST, troponina positiva, pressão arterial sistólica, frequência cardíaca, presença de parada cardíaca ressuscitada e classe de Killip) na admissão hospitalar. Os pacientes com informação ausente sobre uma dessas variáveis não tiveram escore calculado e não foram incluídos neste estudo.

### Análises estatísticas

Números absolutos e porcentagens (%) são relatados para variáveis categóricas. Mediana e percentis (25-75) ou média ± desvio padrão (DP) são usados para descrever variáveis contínuas, de acordo com a distribuição dos dados. O teste de Kolmogorov-Smirnov foi utilizado para avaliar a normalidade dos dados. O teste qui-quadrado foi utilizado para a comparação entre as variáveis categóricas. O teste t de Student não pareado (para distribuição gaussiana) ou o teste de Mann-Whitney (para distribuição não gaussiana) foram utilizados para análise de variáveis contínuas. Para analisar a discriminação de cada escore para predizer óbitos intra-hospitalares, foram construídas curvas ROC para toda a população, e para os subgrupos com IAMCSST e NSTEACS, e suas áreas sob as curvas (AUC) foram comparadas de acordo com o método de Hanley & McNeil.^21^

A associação independente entre os escores analisados e a mortalidade intra-hospitalar foi avaliada por diferentes modelos de regressão logística multivariada:

1. Para toda a população, incluindo 11 variáveis amplamente estudadas na literatura e associadas a desfechos intra-hospitalares: sexo ao nascer, história de hipertensão, diabetes, insuficiência cardíaca, infarto do miocárdio, tabagismo e ICP e, durante a fase intra-hospitalar, choque cardiogênico, infarto do miocárdio com supradesnivelamento do segmento ST, infarto do miocárdio de parede anterior e ICP primária, além do escore GRACE, EuroSCORE II e escore de sangramento A-H como variáveis independentes. Além disso, três modelos adicionais foram desenvolvidos, cada um incorporando as mesmas 11 variáveis, mas com apenas uma pontuação incluída por modelo.
2. Para toda a população, incluindo apenas o escore GRACE, EuroScore II e escore de sangramento A-H.
3. Para pacientes com infarto do miocárdio com supradesnivelamento do segmento ST, incluindo os três escores acima mais o escore TIMI-STEMI.
4. Para pacientes com SCA sem supradesnivelamento do segmento ST, incluindo os três escores (ver item #2) mais o escore TIMI-NSTEACS.

Por fim, para o acompanhamento a longo prazo, os escores foram dicotomizados de acordo com seus valores medianos, e curvas de Kaplan-Meier foram construídas e o teste de log-rank foi utilizado para comparações univariadas; modelos de regressão de Cox ajustados (mesmas variáveis das análises intra-hospitalares foram incluídas) foram utilizados para as análises da associação independente dos escores com a mortalidade por todas as causas.

Um valor de p <0,05 foi considerado estatisticamente significativo para todos os testes. Todas as análises foram realizadas no software SPSS (versão 28, IBM Corporation), exceto para a comparação entre as áreas sob a curva (AUC) das curvas ROC, para a qual foi utilizado o MedCalc Software Ltd.^22^

## Resultados

### Coorte do estudo

A Tabela 1 mostra as características basais de toda a população (N=999) e da população com dados completos de escore (N=504). As duas populações foram semelhantes, exceto por uma maior proporção de infarto do miocárdio de parede anterior em toda a população (23,3% vs 18,7%, p=0,042). Os escores exibem uma semelhança notável em ambas as coortes; por exemplo, as pontuações medianas do GRACE foram 118 e 117, e as pontuações medianas de sangramento A-H foram 15 e 15, respectivamente.

**Tabela 1:** – Características de toda a população e do subgrupo com dados completos de escore*

Variáveis	População inteira (N=999)	População com dados de escores completos (N=504)
Sexo masculino – n/N (%)	702/999 (70,2)	365/504 (72,4)
Idade mediana (25^o^-75^o^ percentis)	64 (56-71)	63 (56,75-70)
História de hipertensão – n/N (%)	775/997 (77,7)	404/504 (80,2)
História de diabetes – n/N (%)	394/996 (39,6)	204/504 (40,5)
História de insuficiência cardíaca – n/N (%)	90/995 (9,0)	54/504 (10,7)
Infarto prévio – n/N (%)	315/996 (31,6)	146/504 (29)
ICP prévia – n/N (%)	195/997 (19,6)	104/504 (20,6)
Choque cardiogênico – n/N (%)	82/892 (9,2)	50/504 (9,9)
Infarto de parede anterior – n/N (%)	230/989 (23,3)	94/503 (18,7)
Infarto com supra de ST – n/N (%)	228/970 (23,5)	99/503 (19,7)
CRM com CEC – n/N (%)	329/383 (85,9)	187/226 (82,7)
Enxerto de mamária interna – n/N (%)	460/493 (93,3)	299/324 (92,3)
≥ 3 enxertos	331/493 (67,1)	212/324 (65,4)
Escore de sangramento A-H mediano (N=639 – 504) – (25^o^-75^o^ percentis)	15 (11-20)	15 (10-20)
Escore GRACE mediano (N=654 – 504) – (25^o^-75^o^percentis)	118 (96,75-140)	117 (97,25-139)
EuroScore II mediano (N=701 – 504) - (25^o^-75^o^percentis)	2,25 (1,39-4,25)	2,22 (1,35-4,30)
Escore TIMI IAMCSST mediano (N=230 - 99) – (25^o^-75^o^percentis)	4 (3-6)	4 (3-6)
Escore TIMI SCASSST (N=654 - 404) – (25^o^-75^o^percentis)	4 (3-4)	4 (3-4)

* Todos os valores de p = NS, exceto para infarto de parede anterior (p = 0,042); A-H = ACUITY-HORIZONS; CEC: circulação extracorpórea; CRM: cirurgia de revascularização do miocárdio; ICP: intervenção coronária percutânea; IAMCSST: Infarto agudo do miocárdio com supra de segmento ST; SCASSST: Síndrome coronária aguda sem supra de segmento ST.

### Mortalidade intra-hospitalar

A Tabela 2 mostra os valores medianos para as duas populações (falecidos ou que permaneceram vivos até a alta hospitalar). Os valores de todos os escores foram significativamente maiores naqueles que morreram no hospital, com valor de p <0,001 para escore de sangramento AH, escore GRACE, EuroSCORE II e escore TIMI-STEMI para toda a população e aqueles com dados de escore completos, com exceção de TIMI-NSTEACS na coorte com dados de escore completo (A, B). A Tabela Suplementar 1 mostra resultados semelhantes, mas considerando os escores como variáveis categóricas (≤ ou acima da mediana), sendo que o escore GRACE apresentou a maior razão de chances (8,52 para toda a população, 9,49 para aqueles com dados de escore completos).

**Tabela 2:** – Comparação entre pacientes vivos ou falecidos durante a fase intra-hospitalar

.			
A) Toda a população			
Variáveis	**Falecidos N=83**	**Sobreviventes N=916**	Valor de p
Escore de sangramento A-H mediano (25^o^-75^o^percentis); N=639	19 (23-36)	14 (10-19)	<0,001
Escore GRACE mediano (25^o^-75^o^percentis); N=654	166,5 (134,25-208,00)	115 (96-134,75)	<0,001
EuroScore II mediano (25^o^-75^o^percentis); N=701	5,45 (2,67-15,24)	2,15 (1,35-3,91)	<0,001
Escore TIMI IAMCSST mediano (25^o^-75^o^percentis); N=230	6 (4-8)	4 (3-5)	<0,001
Escore TIMI SCASSST (25^o^-75^o^percentis); N=654	4 (4-5)	4 (3-4)	0,004
**B) População com dados de escore completos**			
**Variáveis**	**Falecidos N=43**	**Sobreviventes N=461**	**Valor de p**
Escore de sangramento A-H mediano (25^o^-75^o^percentis); N=504	19 (13-26)	14 (10-19)	<0,001
Escore GRACE mediano (25^o^-75^o^percentis); N=504	166 (132-202)	115 (96-133)	<0,001
EuroScore II mediano (25^o^-75^o^percentis); N=504	5,01 (2,39-14,36)	2,10 (1,34-3,90)	<0,001
Escore TIMI IAMCSST mediano (25^o^-75^o^percentis); N=99	7 (4,75-8,00)	4 (3,00-5,50)	0,004
Escore TIMI SCASSST (25^o^-75^o^percentis); N=404	4 (3-4,50)	4 (3-4)	0,119

A-H = ACUITY-HORIZONS; IAMCSST: infarto agudo do miocárdio com supra de segmento ST; SCASSST: síndrome coronária aguda sem supra de segmento ST.

**Figura 1:**
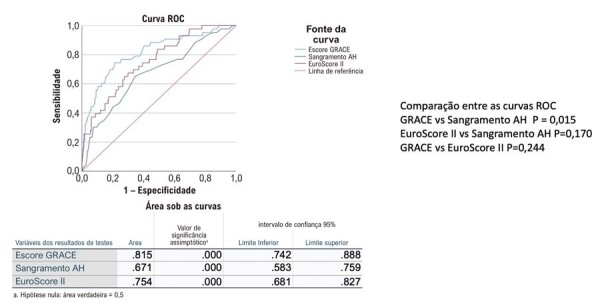
– Capacidade preditiva dos escores de mortalidade intra-hospitalar. Para toda a população, dentre os escores analisados, o GRACE teve o melhor desempenho. A-H: ACUITY HORIZONS; GRACE: Global Registry of Acute Coronary Events.

As análises das curvas ROC na Tabela Suplementar 2 comparam os diferentes escores em relação à AUC, tanto para toda a população quanto para o subconjunto com dados completos de escores. Não foram observadas diferenças estatisticamente significativas entre os conjuntos de dados total e completo para qualquer uma das AUCs obtidas. A Figura 1 e as Figuras Suplementares 1 e 2 exibem as comparações da AUC em todos os subgrupos de pacientes, IAMCSST e SCASSST. O ponto de corte ideal do escore GRACE foi de 136,5 e, ao comparar as diferentes AUCs, o escore GRACE teve melhor desempenho quando comparado ao A-H em toda a população, IAMCSST e NSTEACS; além disso, foi superior ao EuroSCORE II no subgrupo com IAMCSST.

A Tabela 3 mostra as variáveis que foram significativa e independentemente associadas à mortalidade intra-hospitalar nos modelos ajustados. O escore GRACE foi o único escore significativa e independentemente associado à mortalidade em todos os modelos em que foi incluído: um modelo com 14 variáveis independentes (OR=1,030 para cada ponto, p<0,001, Tabela 3A), e modelos com apenas os escores incluídos como variáveis independentes (Tabela 3B) para toda a população, OR=1,026 (p<0,001), para o subgrupo com IAMCSST (OR=1,078; p=0,002), e para o subgrupo com NSTEACS (OR=1,031; p<0,001). Nos três modelos em que testamos um escore de cada vez, o escore GRACE e o EuroSCORE II foram independentemente associados à mortalidade intra-hospitalar (p<0,001 para ambos), mas não o escore de sangramento A-H. Como esperado, o choque cardiogênico foi independentemente associado à mortalidade intra-hospitalar nos três modelos, com valores de p <0,001.

**Tabela 3:** – Variáveis associadas de forma significativa e independente à mortalidade intra-hospitalar

.		
A) Modelo incluindo 11 variáveis independentes além dos escores A-H, GRACE e EuroScore II*		
Variáveis	Valor de p	OR (Intervalo de confiança de 95%)
Choque cardiogênico	<0,001	22,57 (9,35-54,52)
IAMCSST	0,004	6,66 (1,85-23,91)
GRACE score	<0,001	1,03 (1,02-1,04)
*14 Variáveis incluídas no modelo – ver “Métodos”		
**B) Modelos que incluem apenas os escores como variáveis independentes**		
**Variáveis**	**Valor de p**	**OR (Intervalo de confiança de 95%)**
**População inteira**		
GRACE score	<0,001	1,03 (1,02-1,04)*
EuroScore II	0,003	1,11 (1,04-1,19)*
**IAMCSST (N=99)**		
GRACE score	0,002	1,08 (1,03-1,13)*
**SCASSST (N=404)**		
GRACE score	<0,001	1,03 (1,02-1,04)*
EuroScore II	0,010	1,11 (1,03-1,20)*

*Para cada ponto; IAMCSST: Infarto agudo do miocárdio com supra de segmento ST; SCASSST: Síndrome coronária aguda sem supra de segmento ST; OR: odds ratio.

### Mortalidade a longo prazo

A associação entre os escores analisados e a mortalidade em longo prazo na Tabela Suplementar 3 mostra o tempo de sobrevida estimado de Kaplan-Meier para cada um dos escores, representados como variáveis categóricas considerando seus valores medianos (≤mediana ou >mediana). Para toda a população, todos os escores apresentaram taxas de sobrevida significativamente menores para pacientes com escores >mediana, em comparação com aqueles com escores ≤mediana, com valores de p <0,001 para os escores GRACE e TIMI-STEMI. Excluindo-se os óbitos intra-hospitalares, apenas os escores GRACE e TIMI foram significativamente associados à sobrevida, sendo que o escore GRACE apresentou os maiores valores de qui-quadrado em ambas as análises, considerando a totalidade dos óbitos e apenas os óbitos após a alta. As AUCs obtidas a partir das curvas ROC para toda a população foram 0,712 ± 0,035, 0,646 ± 0,037 e 0,643 ± 0,035, respectivamente, para escore GRACE, escore de sangramento A-H e EuroScore II ( Figura Central ). Excluindo-se os óbitos intra-hospitalares, os números foram, respectivamente, 0,638 ± 0,046, 0,613 ± 0,045 e 0,574 ± 0,043.

Os modelos ajustados de correlação com a mortalidade a longo prazo (escores como variáveis contínuas) na Tabela Suplementar 4 mostram as variáveis que foram significativa e independentemente associadas à mortalidade a longo prazo (65 óbitos) dos 314 pacientes com seguimento disponível (seguimento médio 2013 ± 1450 dias). No modelo com as 14 variáveis incluídas (4A), choque cardiogênico (HR=2,737, p=0,006), EuroSCORE II (HR=1,077, p=0,005) e escore GRACE (HR=1,002, p=0,018) foram significativamente associados à mortalidade; excluindo os óbitos intra-hospitalares (19 pacientes), apenas o escore GRACE foi significativamente associado à mortalidade (HR=1,013, p=0,003). No modelo com apenas os escores incluídos (4B), o escore GRACE (HR=1,14, p<0,001) e o EuroSCORE II (HR 1,070, p=0,011) foram significativamente associados à mortalidade em toda a população, e apenas o escore GRACE apresentou associação significativa com a mortalidade em longo prazo (HR=1,013, p=0,005) quando excluídos os óbitos intra-hospitalares.

## Discussão

Em uma população do mundo real com SCA submetida à CRM durante a internação índice, entre os escores de risco isquêmicos, hemorrágicos e cirúrgicos analisados, o melhor desempenho foi obtido pelo escore de risco GRACE.

Anteriormente, um estudo europeu com cerca de 2500 pacientes com SCA submetidos à CRM durante a internação índice^3^ demonstrou uma taxa de mortalidade intra-hospitalar de 8,1%, semelhante aos 8,3% observados na presente publicação. Dos preditores de mortalidade intra-hospitalar, nossa população era quatro anos mais jovem (idade média de 67,8 vs. 63,5 anos, OR referida para idade = 1,03), mas apresentou maior proporção de mulheres (OR relatado = 1,73, incidências de 29,8% vs. 22,4%) e infarto do miocárdio prévio (OR relatado = 1,83, incidências de 31,6% vs. 27,1%). Além disso, nossa população apresentou maior prevalência de diabetes (39,6% vs. 32,1%) e cirurgia cardíaca prévia (5,7% vs. 2,2%), com fração de ejeção do ventrículo esquerdo (50,3% vs. 50,7%) e incidência de IAMCSST (22,8% vs. 23%) similares.

**Escores e mortalidade intra-hospitalar:** Os escores de risco ajudam a identificar pacientes de alto risco que podem se beneficiar de estratégias invasivas precoces ou terapia medicamentosa intensificada, orientam o uso de agentes antitrombóticos contrabalanceando o risco de sangramento e auxiliam na tomada de decisão compartilhada com pacientes e familiares. Publicações anteriores encontraram associação positiva dos escores analisados com a mortalidade em curto prazo.^15 , 17 , 23 - 25^ No presente estudo, todos os escores apresentaram associação significativa com a mortalidade intra-hospitalar quando analisados como variáveis contínuas. Quando analisadas como variáveis categóricas, a maior razão de chances foi obtida com o escore GRACE (OR = 8,52 para toda a população, OR = 9,49 para o subconjunto com dados completos do escore). Além disso, o escore GRACE foi o único escore associado de forma significativa e independente à mortalidade intra-hospitalar em todos os modelos multivariados.

Em relação ao valor preditivo dos diferentes escores, analisados por curvas ROC, estudos anteriores do escore de risco GRACE demonstraram consistentemente medidas de AUC maiores que 0,80.^13 , 22^ Para o escore de sangramento A-H, foi descrito um valor de 0,75;^25^ para TIMI-STEMI, o resultado da publicação original foi de 0,78; para TIMI-NSTEACS, foi encontrado 0,65 para mortalidade por todas as causas, infarto do miocárdio ou revascularização de urgência^16^e 0,60 em publicação subsequente considerando mortalidade em 30 dias.^26^ Finalmente, para o EuroSCORE II, foi relatada uma AUC de 0,81 e em uma ampla população submetida a cirurgia cardíaca de grande porte (47% de revascularização do miocárdio isolada).^17^ De modo geral, os resultados do presente estudo estão de acordo com essas publicações, com AUC de 0,82, 0,67, 0,78, 0,58 e 0,75, respectivamente, para o escore GRACE, escore de sangramento A-H, escore TIMI-STEMI, TIMI-NESTACS e EuroSCORE II.

Publicações anteriores compararam diferentes escores de risco em pacientes com SCA suspeita ou confirmada.^23 , 26 - 30^ No entanto, até onde sabemos, nenhum comparou os escores de risco especificamente em pacientes com SCA submetidos à cirurgia de revascularização miocárdica durante a hospitalização índice, muito menos considerando os escores tradicionais da SCA, juntamente com um escore de sangramento e um escore de risco dedicado para cirurgia cardíaca. As análises da curva ROC mostraram a maior AUC para o escore GRACE; além disso, o escore GRACE foi o único associado de forma significativa e independente à mortalidade intra-hospitalar em todos os modelos ajustados desenvolvidos.

**Escores e mortalidade em longo prazo:** O papel dos vários escores na mortalidade a médio/longo prazo após um episódio de SCA foi descrito anteriormente,^20 , 23 , 27^ mostrando em geral bom desempenho no total. Assim como a mortalidade intra-hospitalar, até onde sabemos, este é o primeiro estudo a comparar diferentes escores de risco para mortalidade em longo prazo em pacientes com SCA submetidos à CRM durante a mesma hospitalização. No presente estudo, as curvas de Kaplan-Meier mostraram o escore GRACE com melhor desempenho entre os escores analisados, sendo o único escore associado de forma significativa e independente à mortalidade em longo prazo em todos os modelos ajustados.

### Limitações

Como todos os estudos observacionais, o nosso tem várias limitações. Primeiro, como em qualquer estudo derivado de um banco de dados retrospectivo, existe a possibilidade de que fatores de confusão não contabilizados nos modelos ajustados possam ter influenciado os resultados. No entanto, a comparação das curvas ROC fornece uma avaliação útil e mais abrangente da hipótese. Em segundo lugar, não tínhamos os dados de todos os escores analisados coletados prospectivamente, e havia muitos com dados ausentes; no entanto, a comparação entre o conjunto de dados completo e o conjunto de dados com todos os escores disponíveis não mostrou diferenças significativas. Em terceiro lugar, nossos resultados foram derivados de um conjunto de dados de centro único e os resultados podem não ser replicáveis para outras instituições. Em quarto lugar, nossos resultados de longo prazo devem ser analisados com cuidado, dada a taxa considerável de perda de seguimento, o que pode introduzir viés e reduzir o poder estatístico para encontrar algumas associações. Em quinto lugar, mudanças no manejo da rotina, incluindo melhorias no tratamento médico e nas técnicas de ICP e CRM durante o período, bem como critérios de seleção de estratégias de revascularização coronariana, podem ter influenciado nossos resultados. É importante ressaltar que nossos protocolos institucionais foram atualizados constantemente de acordo com as diretrizes nacionais e internacionais. Por fim, não foi possível recuperar o escore STS e ele não foi analisado no presente estudo; no entanto, é importante notar que a comparação entre STS e EuroSCORE II em populações brasileiras e indianas mostrou resultados semelhantes.^31 , 32^

## Conclusão

Em pacientes com SCA submetidos à CRM durante a internação-índice, o escore GRACE foi o único escore de risco que permaneceu independentemente associado à mortalidade intra-hospitalar e em longo prazo em todos os modelos desenvolvidos, após ajuste para potenciais fatores de confusão. Além disso, o escore GRACE apresentou o melhor desempenho na predição de óbitos intra-hospitalares. Esses achados podem influenciar o processo de tomada de decisão clínica nessa população de alto risco.

## Material suplementar

Material suplementar
