# Supplementary material for: Comparação entre Cinco Escores de Risco em Pacientes com Síndromes Coronárias Agudas Submetidos à Revascularização Cirúrgica durante a Internação Índice
Source: Arq Bras Cardiol. 2025 Oct 8;122(11):e20250320. [Article in Portuguese] doi: 10.36660/abc.20250320 (PMC12711224; doi:10.36660/abc.20250320)
Supplement: Material suplementar [file 0066-782x-abc-122-11-e20250320-suppl01.pdf]

Material suplementar

Tabela Suplementar 1: Associação entre os escores analisados (como variáveis categóricas) e mortalidade intra-hospitalar

A) Toda a população

Variáveis	Falecidos N=83	Sobreviventes N=916	Valor de p	OR (IC 95%)
Escore de sangramento AH – % acima da mediana; N=639	12.3	5.3	0.002	2.49 (1.39-4.48)
Escore GRACE – % acima da mediana; N=654	15.5	2.1	<0,001	8.52 (3.81-19.08)
EuroScore II – % acima da mediana; N=701	12.6	3.4	<0,001	4.06 (2.11-7.83)
Escore TIMI IAMCSST – acima da mediana; N=230	17.2	5.3	0.004	3.67 (1.46-9.25)
Escore TIMI SCASSST – % acima da mediana; N=654	9.3	6.7	0.296	1.43 (0.76-2.71)

B) População com dados de pontuação completos

Variáveis	Falecidos N=43	Sobreviventes N=461	Valor de p	OR IC 95%)
Escore de sangramento AH – % acima da mediana; N=504	12.6	4.9	0.002	2.81 (1.43-5.52)
Escore GRACE – % acima da mediana; N=504	15.6	1.9	<0,001	9.49 (3.67-24.55)
EuroScore II – % acima da mediana; N=504	13.3	3.9	<0,001	3.78 (1.82-7.84)
Escore TIMI IAMCSST - % acima da mediana; N=99	17.0	3.8	0.028	5.23 (1.05-26.03)
Escore TIMI SCASSST – % acima da mediana; N=404	8.2	8.2	0.992	1.00 (0.43-2.28)

Tabela suplementar 2: Comparações da curva ROC (mortalidade intra-hospitalar)

**Variáveis**	**População total (N=999)**	**População com dados de pontuação completa (N=504)**	**Valor de p**
Escore de sangramento AH (AUC ± EP)	0,658 ± 0,041; p<0,001 (N=639)	0,671 ± 0,045; p<0,001 (N=504)	0.79
Escore GRACE (AUC ± E.P.)	0,805 ± 0,034; p<0,001 (N=654)	0,815 ± 0,037; p<0,001 (N=504)	0.84
EuroScore II (AUC ± EP)	0,768 ± 0,033; p<0,001 (N=701)	0,754 ± 0,037; p<0,001 (N=504)	0.78
Escore TIMI IAMCSST (AUC ± EP)	0,738 ± 0,057; p<0,001 (N=230)	0,780 ± 0,077; p=0,004 (N=99)	0.70
Escore TIMI SCASSST (AUC ± EP)	0,620 ± 0,038; p=0,006 (N=654)	0,579 ± 0,046; p=0,131 (N=404)	0.49

A-H=ACUITY-HORIZONS; AUC = área sob a curva; EP = erro padrão; IAMCSST = Infarto agudo do miocárdio com supra de segmento ST; SCASSST = Síndrome coronária aguda sem supra de segmento ST

Tabela Suplementar 3: Tempos de sobrevida estimados pelo método de Kaplan Meyer (dias)

A) Toda a população

Variáveis	Média ± DP	Qui-Quadrado/Log-Rank
Escore GRACE ≤mediana/>mediana	4604,90 ± 143,85/3323,59 ± 166,44	32,65/<0,001
Escore de sangramento AH ≤mediana/>mediana	3913,75 ± 172,28/3388,11 ± 195,93	5.97/0.015
EuroScore II ≤mediana/>mediana	3822,54 ± 164,77/3452,07 ± 206,19	4.90/0.027
TIMI- SCASSST ≤mediana/>mediana	4112,67 ± 144,41/3480,06 ± 263,95	5.42/0.020
TIMI- IAMCSST ≤mediana/>mediana	4473,44 ± 279,14/2523,04 ± 335,96	15,23/<0,001

B) Excluindo óbitos intra-hospitalares

Variáveis	Média ± DP	Qui-Quadrado/Log-Rank
Escore GRACE ≤mediana/>mediana	4682,21 ± 141,07/3816,76 ± 168,01	15.27/<0.001
Escore de sangramento AH ≤mediana/>mediana	4080,59 ± 170,10/3732,09 ± 199,12	2.84/0.092
EuroScore II ≤mediana/>mediana	3938,63 ± 163,41/3872,90 ± 210,37	0.58/0.445
TIMI- SCASSST ≤mediana/>mediana	4342,66 ± 141,83/3842,51 ± 267,32	3.35/0.067
TIMI- IAMCSST ≤mediana/>mediana	4581,33 ± 246,38/3147,71 ± 350,73	12,58/<0,001

Tabela Suplementar 4: Variáveis associadas de forma significativa e independente à mortalidade a longo prazo

A) População total (N=314) com 11 variáveis independentes além do escore de sangramento A-H, escore GRACE e EuroScore II

Variáveis	Valor de p	HR (intervalo de confiança de 95%)
Choque cardiogênico	0.006	2.74 (1.33-5.65)
EuroScore II	0.005	1.08 (1.02-1.13)
Escore GRACE	0.018	1.01 (1.00-1.02)

A1) Excluindo óbitos intra-hospitalares (N=281)

Variáveis	Valor de p	HR (intervalo de confiança de 95%)
Escore GRACE	0.003	1.01 (1.00-1.02)

B) População total com apenas os escores como variáveis independentes

Variáveis	Valor de p	HR (intervalo de confiança de 95%)
EuroScore II	0.011	1.07 (1.02-1.13)
Escore GRACE	<0,001	1.01 (1.01-1.02)

B1) Excluindo óbitos intra-hospitalares

Variáveis	Valor de p	HR (intervalo de confiança de 95%)
Escore GRACE	0.005	1.01 (1.00-1.02)

Figura suplementar 1: Capacidade preditiva dos escores de mortalidade intra-hospitalar em pacientes com IAMCSST

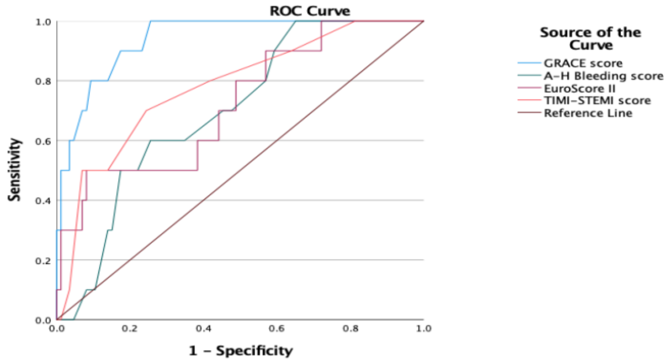

Area Under the Curve

Test Result Variable(s)	Area	Std. Error[a]	Asymptotic Sig.[b]	Asymptotic 95% Confidence Interval	
				Lower Bound	Upper Bound
GRACE score	.940	.029	.000	.882	.997
A–H Bleeding score	.700	.073	.039	.557	.843
EuroScore II	.722	.086	.022	.553	.891
TIMI STEMI score	.780	.077	.004	.628	.931

a. Under the nonparametric assumption

b. Null hypothesis: true area = 0.5

Comparison between the ROC curves

- A-H Bleeding vs TIMI STEMI P=0.451
- GRACE vs TIMI STEMI P=0.052
- EuroScore II vs TIMI STEMI P=0.615
- GRACE vs A-H Bleeding P=0.002
- EuroScore II vs A-H Bleeding P=0.845
- GRACE vs EuroScore II P=0.016

Legends as in Table 1

Figura suplementar 2: Capacidade preditiva dos escores de mortalidade intra-hospitalar em pacientes com SCASSST

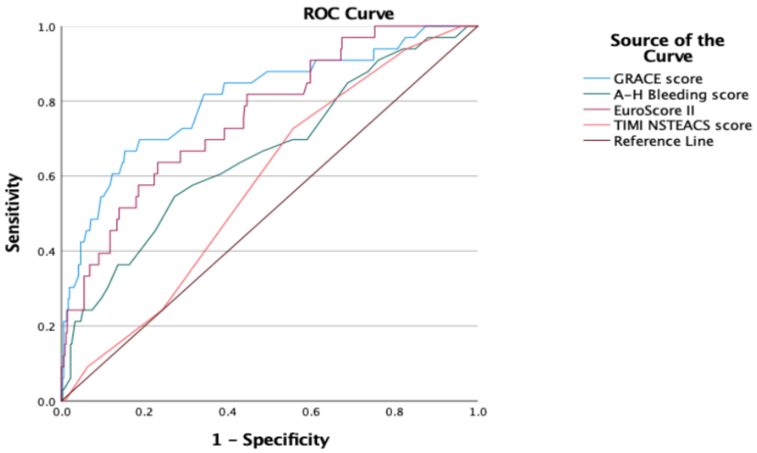

Area Under the Curve

Test Result Variable(s)	Area	Std. Error[a]	Asymptotic Sig.[b]	Asymptotic 95% Confidence Interval	
				Lower Bound	Upper Bound
GRACE score	.806	.044	.000	.719	.892
A-H Bleeding score	.659	.052	.002	.557	.762
EuroScore II	.760	.043	.000	.676	.844
TIMI NSTEACS score	.579	.046	.134	.489	.669

a. Under the nonparametric assumption

b. Null hypothesis: true area = 0.5

Comparison between the ROC curves

- A-H Bleeding vs TIMI NSTEMI P=0.249
- GRACE vs TIMI NSTEMI P<0.001
- EuroScore II vs TIMI NSTEMI P=0.004
- GRACE vs A-H Bleeding P=0.031
- EuroScore II vs A-H Bleeding P=134
- GRACE vs EuroScore II P=0.455

Legends as in Table 1
