# Supplementary material for: Comparação entre Cinco Escores de Risco em Pacientes com Síndromes Coronárias Agudas Submetidos à Revascularização Cirúrgica durante a Internação Índice
Source: Arq Bras Cardiol. 2025 Oct 8;122(11):e20250320. [Article in Portuguese] doi: 10.36660/abc.20250320 (PMC12711224; doi:10.36660/abc.20250320)
Supplement: SUPPLEMENTAL MATERIAL [file 0066-782x-abc-122-11-e20250320-suppl01-en.pdf]

Supplemental material

Supplemental Table 1: Comparison between patients alive or deceased during the in-hopital phase (scores as categorical variables)".

A) Entire population

Variables	Deceased N=83	Alive N=916	P-value	OR (95% CI)
A-H Bleeding score – % above the median; N=639	12.3	5.3	0.002	2.49 (1.39-4.48)
GRACE score – % above the median; N=654	15.5	2.1	<0.001	8.52 (3.81-19.08)
EuroScore II – % above the median; N=701	12.6	3.4	<0.001	4.06 (2.11-7.83)
TIMI STEMI score – above the median; N=230	17.2	5.3	0.004	3.67 (1.46-9.25)
TIMI NSTEACS score – % above the median; N=654	9.3	6.7	0.296	1.43 (0.76-2.71)

B) Population with complete score data

Variables	Deceased N=43	Alive N=461	P-value	OR 95% CI)
A-H Bleeding score – % above the median; N=504	12.6	4.9	0.002	2.81 (1.43-5.52)
GRACE score – % above the median; N=504	15.6	1.9	<0.001	9.49 (3.67-24.55)
EuroScore II – % above the median; N=504	13.3	3.9	<0.001	3.78 (1.82-7.84)
TIMI STEMI score - % above the median; N=99	17.0	3.8	0.028	5.23 (1.05-26.03)
TIMI NSTEACS score – % above the median; N=404	8.2	8.2	0.992	1.00 (0.43-2.28)

Supplemental Table 2: ROC curve (in-hospital mortality) comparisons

Variables	Entire population (N=999)	Population with complete score data (N=504)	P-value
A-H Bleeding score (AUC ± SE)	0.658 ± 0.041; p<0.001 (N=639)	0.671 ± 0.045; p<0.001 (N=504)	0.79
GRACE score (AUC ± SE)	0.805 ± 0.034; p<0.001 (N=654)	0.815 ± 0.037; p<0.001 (N=504)	0.84
EuroScore II (AUC ± SE)	0.768 ± 0.033; p<0.001 (N=701)	0.754 ± 0.037; p<0.001 (N=504)	0.78
TIMI STEMI score (AUC ± SE)	0.738 ± 0.057; p<0.001 (N=230)	0.780 ± 0.077; p=0.004 (N=99)	0.70
TIMI non-STEMI score (AUC ± SE)	0.620 ± 0.038; p=0.006 (N=654)	0.579 ± 0.046; p=0.131 (N=404)	0.49

A-H=ACUITY-HORIZONS; AUC = area under curve; STEMI=ST-Elevation MI; NSTEACS=non-ST-Elevation Acute Coronary Syndrome; SD=Standard Deviation

Supplemental Table 3: Kaplan Meyer estimated survival times (days)

A) Entire population

Variables	Mean ± SD	Chi-Square/Log-Rank
GRACE score ≤median/>median	4604.90 ± 143.85/3323.59 ± 166.44	32.65/<0.001
A-H bleeding score ≤median/>median	3913.75 ± 172.28/3388.11 ± 195.93	5.97/0.015
EuroScore II ≤median/>median	3822.54 ± 164.77/3452.07 ± 206.19	4.90/0.027
TIMI-NSTEACS ≤median/>median	4112.67 ± 144.41/3480.06 ± 263.95	5.42/0.020
TIMI-STEMI ≤median/>median	4473.44 ± 279.14/2523.04 ± 335.96	15.23/<0.001

B) Excluding in-hospital deaths

Variables	Mean ± SD	Chi-Square/Log-Rank
GRACE score ≤median/>median	4682.21 ± 141.07/3816.76 ± 168.01	15.27/<0.001
A-H bleeding score ≤median/>median	4080.59 ± 170.10/3732.09 ± 199.12	2.84/0.092
EuroScore II ≤median/>median	3938.63 ± 163.41/3872.90 ± 210.37	0.58/0.445
TIMI-NSTEACS ≤median/>median	4342.66 ± 141.83/3842.51 ± 267.32	3.35/0.067
TIMI-STEMI ≤median/>median	4581.33 ± 246.38/3147.71 ± 350.73	12.58/<0.001

Supplemental Table 4: Variables associated significantly and independently with long-term mortality

A) Entire population (N=314) with 11 independent variables besides A-H Bleeding score, GRACE score and EuroScore II

Variables	P-value	HR (95% confidence interval)
Cardiogenic shock	0.006	2.74 (1.33-5.65)
EuroScore II	0.005	1.08 (1.02-1.13)
GRACE score	0.018	1.01 (1.00-1.02)

A1) Excluding in-hospital deaths (N=281)

Variables	P-value	HR (95% confidence interval)
GRACE score	0.003	1.01 (1.00-1.02)

B) Entire population with only the scores as independent variables

Variables	P-value	HR (95% confidence interval)
EuroScore II	0.011	1.07 (1.02-1.13)
GRACE score	<0.001	1.01 (1.01-1.02)

B1) Excluding in-hospital deaths

Variables	P-value	HR (95% confidence interval)
GRACE score	0.005	1.01 (1.00-1.02)

Supplemental Figure 1: Predictive capacity of the scores for in-hospital mortality in patients with STEMI

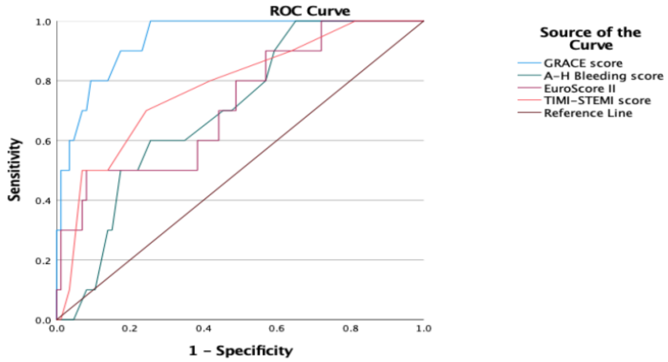

Area Under the Curve

Test Result Variable(s)	Area	Std. Error[a]	Asymptotic Sig.[b]	Asymptotic 95% Confidence Interval	
				Lower Bound	Upper Bound
GRACE score	.940	.029	.000	.882	.997
A–H Bleeding score	.700	.073	.039	.557	.843
EuroScore II	.722	.086	.022	.553	.891
TIMI STEMI score	.780	.077	.004	.628	.931

a. Under the nonparametric assumption

b. Null hypothesis: true area = 0.5

Comparison between the ROC curves

- A-H Bleeding vs TIMI STEMI P=0.451
- GRACE vs TIMI STEMI P=0.052
- EuroScore II vs TIMI STEMI P=0.615
- GRACE vs A-H Bleeding P=0.002
- EuroScore II vs A-H Bleeding P=0.845
- GRACE vs EuroScore II P=0.016

Legends as in Table 1

Supplemental Figure 2: Predictive capacity of the scores for in-hospital mortality in patients with NSTEACS

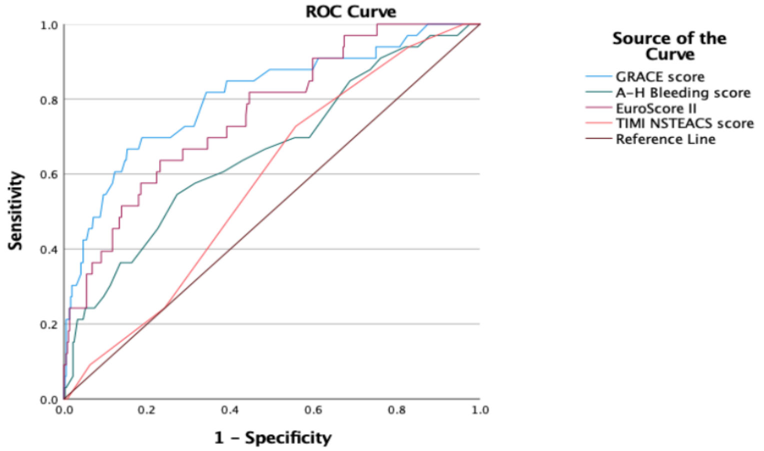

Area Under the Curve

Test Result Variable(s)	Area	Std. Error[a]	Asymptotic Sig.[b]	Asymptotic 95% Confidence Interval	
				Lower Bound	Upper Bound
GRACE score	.806	.044	.000	.719	.892
A-H Bleeding score	.659	.052	.002	.557	.762
EuroScore II	.760	.043	.000	.676	.844
TIMI NSTEACS score	.579	.046	.134	.489	.669

a. Under the nonparametric assumption

b. Null hypothesis: true area = 0.5

Comparison between the ROC curves

- A-H Bleeding vs TIMI NSTEMI P=0.249
- GRACE vs TIMI NSTEMI P<0.001
- EuroScore II vs TIMI NSTEMI P=0.004
- GRACE vs A-H Bleeding P=0.031
- EuroScore II vs A-H Bleeding P=134
- GRACE vs EuroScore II P=0.455

Legends as in Table 1
