# Supplementary material for: Comparação entre Cinco Escores de Risco em Pacientes com Síndromes Coronárias Agudas Submetidos à Revascularização Cirúrgica durante a Internação Índice
Source: Arq Bras Cardiol. 2025 Oct 8;122(11):e20250320. [Article in Portuguese] doi: 10.36660/abc.20250320 (PMC12711224; doi:10.36660/abc.20250320)
Supplement: Translation

# Translation: Comparison between Five Risk Scores in Patients with Acute Coronary Syndromes Undergoing Surgical Revascularization during the Index Hospitalization

**DOI:** 10.36660/abc.20250320i

**Authors:** José C. Nicolau, Roberto R. C. V. Giraldez, Fabio B. Jatene, Luís A. O. Dallan, Luiz A. Lisboa, Omar A. V. Mejia, Jorge L. M. Ribera, Adriadne J. Bertolin, Luciano M. Baracioli, Felipe G. Lima, Maria C. D. Andrade, Santiago A. C. Vintimilla, Leonardo Salis, Fabiane L. de Freitas, Maxim Goncharov, Lucas C. Godoy, Remo H. de M. Furtado, Michael E. Farkouh

**Affiliations:** 1 Brazil Instituto do Coração do Hospital das Clínicas da Faculdade de Medicina da Universidade de São Paulo, São Paulo, SP – Brazil; 2 Canada University of Toronto – Peter Munk Cardiac Centre, Toronto – Canada; 3 USA Cedars-Sinai Health System, Los Angeles, Califórnia – USA

**Keywords:** Acute Coronary Syndrome, Mortality, Myocardial Revascularization, Continuity of Patient Care

## Abstract

**Background:**

Risk scores (RS) for patients with acute coronary syndromes (ACS) and coronary artery bypass grafting (CABG) have been tested previously, but little is known about their value in ACS patients undergoing CABG during the index hospitalization.

**Objectives:**

To compare five different RS in ACS patients undergoing CABG during the index hospitalization.

**Methods:**

The analyzed RS were GRACE, TIMI-non-ST-elevation ACS (TIMI-NSTEACS), TIMI-ST-elevation acute myocardial infarction (TIMI-STEMI), ACUITY/HORIZONS (A-H) bleeding, and EuroSCORE II. The RS were evaluated regarding their performance during the in-hospital phase and the long-term follow-up after discharge; a p-value <0.05 was considered significant.

**Results:**

A total of 999 patients were included between 1998 and 2022. The mean time from symptom onset to CABG was 6.3 ± 5.5 days. The areas under the ROC curves were 0.82 (95% CI 0.74 – 0.89, p<0.001) for GRACE, 0.78 (95% CI 0.62-0.93, P=0.004) for TIMI-STEMI, 0.75 (95% CI 0.61-0.83, p<0.001) for EuroSCORE II, 0.67 (95% CI 0.59-0.76, P<0.001) for A-H bleeding, and 0.58 (95% CI 0.49-0.67, p=0.131) for TIMI-NSTEACS. Excluding in-hospital deaths, only GRACE and TIMI-STEMI were significantly associated with long-term mortality (mean follow-up of 5.5 ± 4 years). In the multivariable analyses, the GRACE score was the only RS significantly associated with in-hospital and long-term mortality in all adjusted models.

**Conclusion:**

In patients with ACS who underwent CABG during the index hospitalization, the GRACE score was the only risk score that remained independently associated with both in-hospital and long-term mortality across all developed models, after adjustment for potential confounders. Moreover, the GRACE score performed better than others in predicting in-hospital deaths. These findings may influence the clinical decision-making process in this high-risk population.

## Introduction

Cardiovascular disease is the leading cause of death worldwide, with ischemic heart disease being the most prevalent among them.^1^In patients with acute coronary syndromes (ACS), around 40% have multivessel coronary artery disease.^2^Despite a decrease in the utilization rates of coronary surgical revascularization, mainly related to the widespread use of percutaneous coronary intervention (PCI), up to 10%-15% of those patients are referred to coronary artery bypass grafting surgery (CABG) during the index ACS hospitalization,^3 - 5^with some evidence of benefit of CABG over PCI in this population, especially in patients with diabetes.^6 - 10^In the US, approximately 35% of the patients with diabetes and non-ST-elevation acute coronary syndromes (NSTEACS) undergo CABG during the index hospitalization.^11^

Cardiovascular scores in general have limitations.^12^Different scores have been developed for risk stratification in patients with ACS, evaluating either bleeding (ACUITY/HORIZONS bleeding score)^13^or ischemic events (GRACE score),^14^in patients with ST-elevation myocardial infarction (TIMI-STEMI score)^15^or with NSTEACS (TIMI-NSTEACS score).^16^Additionally, cardiac risk scores to predict mortality post-major cardiac surgery, such as the EuroSCORE II, have been validated.^17^Interestingly, a higher proportion of PCI utilization, in comparison with CABG (higher PCI: CABG rate), seems to be associated with an increased risk for CABG-associated in-hospital mortality among ACS patients.^5^

To the best of our knowledge, these risk scores have not been assessed in a real-world, non-selected population of patients with ACS undergoing CABG during the index hospitalization. This study aims to compare the performance of various risk scores in ACS patients undergoing CABG during the index hospitalization, focusing on in-hospital all-cause mortality. The main secondary analysis was the comparison between the scores in the long-term after discharge in the population with available data.

## Materials and methods

### Compliance with ethical standards

This study conformed to the International Council on Harmonization norms on medical research in humans. The Ethics Committee of Clinics Hospital approved the study. Because the data are based on individual information obtained for administrative purposes, informed consent was waived, according to local regulations.

### Population

From an administrative databank collected prospectively at the Heart Institute, University of São Paulo, an academic tertiary medical center, we retrospectively analyzed 999 consecutive patients admitted with ACS at the Coronary Care Unit (median age 64 years, 70.2% males) who underwent CABG during the index hospitalization from 1999 to 2022. Supplemental Figure 1 summarizes the inclusions throughout this period. The mean time from symptom onset to CABG was 6.3 ± 5.5 days. All patients were taking dual antiplatelet therapy. Except for 95 patients participating in a randomized study where a strategy of timing to send the patient to CABG was based on platelet function assessment,^18^the waiting time from P2Y12 discontinuation and CABG was 5-7 days, following guidelines recommendation.^19^STEMI patients were often initially treated with fibrinolytic therapy in the referring institutions before being transferred to our center (mean time 1.04 ± 1.83 days). Data collected included demographic variables, as well as index-event presentation characteristics. Regarding the long-term follow-up, even though our databank was locked in 2022, the last attempt to contact all patients was carried out in 2015 (the maximum follow-up time was 16 years, with a mean of 5.52 years), and 249 patients were included between 2015 and 2022, corresponding to 24.9% of the patients lost to follow-up. At the end, we got the survival status of 597 patients for the entire population (59.76%) and 314 patients for the population with complete score data (62.3%).

**Figure 1:** – Predictive capacity of the scores for in-hospital mortality. For the entire population, among the analyzed scores, the GRACE had the best performance. A-H: ACUITY HORIZONS; GRACE: Global Registry of Acute Coronary Events.

### Scores

All analyzed scores are widely used and have been validated in various publications.^16 , 17 , 20^Given the time span of the data collection, some of the scores data were collected retrospectively, being the EuroSCORE II data^17^and other surgical variables collected entirely retrospectively from patients’ charts and other hospital sources by two of the authors (FLF, OAVM), and added to the original databank. Regarding the A-H bleeding score, given that bivalirudin is not available in the country, it was always computed as zero in the score. For the GRACE score, it was used the version 2.0.^20^The score was calculated based on information collected in our databank, which includes the required information to calculate the score (age, creatinine, ST-segment deviation, positive troponin, systolic blood pressure, heart rate, presence of resuscitated cardiac arrest, and Killip class) at hospital admission. Patients with missing information on one of these variables did not have a score calculated and were not included in this study.

### Statistical analyses

Absolute numbers and percentages (%) are reported for categorical variables. Median and percentiles (25^th^-75th) or mean ± standard deviation (SD) are used to describe continuous variables, according to the distribution of the data. The Kolmogorov-Smirnov test was utilized to assess data normality. The chi-square test was utilized for the comparison between categorical variables. The unpaired Student’s t-test (for Gaussian distribution) or Mann-Whitney test (for non-Gaussian distribution) was utilized to analyze continuous variables. To analyze the discrimination of each score to predict in-hospital deaths, ROC curves were constructed for the entire population, and for the subgroups with STEMI and NSTEACS, and their area under the curves (AUC) were compared according to the Hanley & McNeil method.^21^

Independent association between the analyzed scores and in-hospital mortality was evaluated by different multivariable logistic regression models:

For the entire population, including 11 variables extensively studied in the literature and associated with in-hospital outcomes: at baseline sex, history of hypertension, diabetes, heart failure, myocardial infarction, smoking, and percutaneous coronary intervention – PCI and during the in-hospital phase cardiogenic shock, ST-Elevation Myocardial Infarction, anterior-wall myocardial infarction and primary PCI, besides GRACE score, EuroSCORE II and A-H bleeding score as independent variables. Furthermore, three additional models were developed, each incorporating the same eleven variables, but with only one score included per model.

For the entire population, including only the GRACE score, EuroScore II, and A-H bleeding scores.

For patients with ST-elevation MI, including the three scores above, plus the TIMI-STEMI score.

For patients with non-ST-elevation ACS, including the three scores (see item #2) plus the TIMI-NSTEACS score.

Finally, for the long-term follow-up, the scores were dichotomized according to their median values, Kaplan-Meier curves were constructed, and the log-rank test was utilized for univariate comparisons; adjusted Cox regression models (same variables as the in-hospital analyses were included) were used for the analyses of the independent association of the scores with all-cause mortality.

A p-value <0.05 was considered statistically significant for all tests. All analyses were performed using the SPSS software (version 28, IBM Corporation), except for the comparison between the area under the curve (AUC) of the ROC curves, for which we utilized the MedCalc Software Ltd.^22^

## Results

### Study cohort

Table 1 shows the baseline characteristics of the entire population (N=999) and the population with complete score data (N=504). The two populations were similar, except for a higher proportion of anterior-wall myocardial infarction in the entire population (23.3% vs 18.7%, P=0.042). The scores display a remarkable similarity in both cohorts; for instance, the median GRACE scores were 118 and 117, and the median A-H bleeding scores were 15 and 15, respectively.

**Table 1:** – Characteristics of the entire population and the subgroup with complete score data*

Variables	Entire population (N=999)	Population with complete score data (N=504)
Male sex – n/N (%)	702/999 (70.2)	365/504 (72.4)
Median age (25th- 75th percentiles)	64 (56-71)	63 (56.75-70)
History of hypertension – n/N (%)	775/997 (77.7)	404/504 (80.2)
History of diabetes – n/N (%)	394/996 (39.6)	204/504 (40.5)
History of heart failure – n/N (%)	90/995 (9.0)	54/504 (10.7)
Previous MI – n/N (%)	315/996 (31.6)	146/504 (29)
Previous PCI – n/N (%)	195/997 (19.6)	104/504 (20.6)
Cardiogenic shock – n/N (%)	82/892 (9.2)	50/504 (9.9)
Anterior wall MI – n/N (%)	230/989 (23.3)	94/503 (18.7)
ST-elevation MI – n/N (%)	228/970 (23.5)	99/503 (19.7)
On-pump CABG – n/N (%)	329/383 (85.9)	187/226 (82.7)
Left mammary graft – n/N (%)	460/493 (93.3)	299/324 (92.3)
≥ 3 grafts	331/493 (67.1)	212/324 (65.4)
Median A-H bleeding score (N=639 – 504) – (25th- 75th percentiles)	15 (11-20)	15 (10-20)
Median GRACE score (N=654 – 504) – (25th- 75th percentiles)	118 (96.75-140)	117 (97.25-139)
Median EuroScore II (N=701 – 504) - (25th- 75th percentiles); mean (± SD)	2.25 (1.39-4.25)	2.22 (1.35-4.30)
Median TIMI STEMI score (N=230 - 99) – (25th- 75th percentiles)	4 (3-6)	4 (3-6)
Median TIMI NSTEACS score (N=654 - 404) – (25th- 75th percentiles)	4 (3-4)	4 (3-4)

* All p-values=NS except for anterior-wall MI (p=0.042); MI: myocardial infarction; PCI: percutaneous coronary intervention; A-H=ACUITY-HORIZONS; STEMI: ST-Elevation MI; NSTEACS: non-ST-Elevation Acute Coronary Syndrome; SD: Standard Deviation.

### In-hospital mortality

Table 2 shows the median values for the two populations (deceased or discharged alive). The values of all scores were significantly higher in those who died in-hospital, with p-value <0.001 for A-H bleeding score, GRACE score, EuroSCORE II, and TIMI-STEMI score for the entire population and those with complete score data, except for TIMI-NSTEACS in the cohort with complete score data (A, B). Supplemental Table 1 shows similar results but considering the scores as categorical variables (≤ or above the median), with the GRACE score having the highest odds ratio (8.52 for the entire population, 9.49 for those with complete score data).

**Table 2:** – Comparison between patients alive or deceased during the in-hospital phase

.			
A) Entire population			
Variables	**Deceased N=83**	**Alive N=916**	p-value
A-H Bleeding score – median (25^th^- 75^th^percentiles); N=639	19 (23-36)	14 (10-19)	<0.001
GRACE score – median (25^th^- 75^th^percentiles); N=654	166.5 (134.25-208.00)	115 (96-134.75)	<0.001
EuroScore II – median (25^th^- 75^th^percentiles); N=701	5.45 (2.67-15.24)	2.15 (1.35-3.91)	<0.001
TIMI STEMI score – median (25^th^- 75^th^percentiles); N=230	6 (4-8)	4 (3-5)	<0.001
TIMI NSTEACS score – median (25^th^- 75^th^percentiles); N=654	4 (4-5)	4 (3-4)	0.004
**B) Population with complete score data**			
**Variables**	**Deceased N=43**	**Alive N=461**	**p-value**
A-H Bleeding score – median (25^th^- 75^th^percentiles); N=504	19 (13-26)	14 (10-19)	<0.001
GRACE score – median (25^th^- 75^th^percentiles); N=504	166 (132-202)	115 (96-133)	<0.001
EuroScore II – median (25^th^- 75^th^percentiles); N=504	5.01 (2.39-14.36)	2.10 (1.34-3.90)	<0.001
TIMI STEMI score – median (25^th^- 75^th^percentiles); N=99	7 (4.75-8.00)	4 (3.00-5.50)	0.004
TIMI NSTEACS score – median (25^th^- 75^th^percentiles); N=404	4 (3-4.50)	4 (3-4)	0.119

A-H: ACUITY-HORIZONS; STEMI: ST-Elevation MI; NSTEACS: non-ST-Elevation Acute Coronary Syndrome; SD: Standard Deviation.

ROC curve analyses in Supplemental Table 2 compare the different scores regarding the AUC, both for the entire population and for the subset with complete score data. No statistically significant differences were observed between the total and complete datasets for any of the obtained AUCs. Figure 1 and Supplemental Figures 1 and 2 display the AUC comparisons across all patients, STEMI, and NSTEACS subgroups. The optimal GRACE score cut-off was 136.5, and, when comparing the different AUCs, the GRACE score performed better when compared to A-H bleeding in the entire population, STEMI, and NSTEACS; additionally, it was superior to EuroSCORE II in the subgroup with STEMI.

Table 3 shows the variables that were significantly and independently associated with in-hospital mortality in the adjusted models. GRACE score was the only score significantly and independently associated with mortality in all models were it was included: a model with 14 independent variables (OR=1.030 for each point, p<0.001, Table 3A), and models with only the scores included as independent variables (Table 3B) for the entire population, OR=1.026 (P<0.001), for the subgroup with STEMI, OR=1.078 (p=0.002), and for the subgroup with NSTEACS, OR=1.031 (p<0.001. In the three models where we tested one score at a time, the GRACE score and EuroSCORE II were independently associated with in-hospital mortality (p<0.001 for both), but not the A-H bleeding score. As expected, cardiogenic shock was independently associated with in-hospital mortality in the three models, with p-values of <0.001.

**Table 3:** – Variables associated significantly and independently with in-hospital mortality

.		
A) Model including 11 independent variables besides A-H Bleeding score, GRACE score, and EuroScore II*		
Variables	p-value	OR (95% confidence interval)
Cardiogenic shock	<0.001	22.57 (9.35-54.52)
STEMI	0.004	6.66 (1.85-23.91)
GRACE score	<0.001	1.03 (1.02-1.04)
*14 variables included in the model – see “methods”		
**B) Models including only the scores as independent variables**		
**Variables**	**p-value**	**OR (95% confidence interval)**
**Entire population**		
GRACE score	<0.001	1.03 (1.02-1.04)*
EuroScore II	0.003	1.11 (1.04-1.19)*
**STEMI (N=99)**		
GRACE score	0.002	1.08 (1.03-1.13)*
**NSTEACS (N=404)**		
GRACE score	<0.001	1.03 (1.02-1.04)*
EuroScore II	0.010	1.11 (1.03-1.20)*

*For each point; STEMI: ST-Elevation Myocardial Infarction; NSTEACS: non-ST-Elevation Acute Coronary Syndrome; OR: odds ratio.

### Long-term mortality

The association between the analyzed scores and long-term mortality in Supplemental Table 3 shows the Kaplan-Meier estimated survival time for each of the scores, represented as categorical variables considering their median values (≤median or >median). For the entire population, all scores showed significantly lower survival rates for patients with >median scores, in comparison with those with ≤median scores, with p-values <0.001 for GRACE and TIMI-STEMI scores. Excluding in-hospital deaths, only GRACE and TIMI-scores were significantly associated with survival, and the GRACE score showed the highest chi-square values in both analyses, considering the totality of deaths and only deaths after discharge. The AUCs obtained from ROC curves for the entire population were 0.712 ± 0.035, 0.646 ± 0.037 and 0.643 ± 0.035, respectively, for GRACE score, A-H bleeding score, and EuroScore II ( Central Illustration ). Excluding in-hospital deaths, the figures were, respectively, 0.638 0.046, 0.613 ± 0.045 and 0.574 ± 0.043.

Adjusted models for correlation with long-term mortality (scores as continuous variables) in Supplemental Table 4 show the variables that were significantly and independently associated with long-term mortality (65 deaths) from the 314 patients with available follow-up (mean follow-up 2013 ± 1450 days). In the model with the 14 variables included (4A), cardiogenic shock (HR=2.737, p=0.006), EuroSCORE II (HR=1.077, p=0.005), and GRACE score (HR=1.002, p=0.018) were significantly associated with mortality; excluding in-hospital deaths (19 patients), only the GRACE score was significantly associated with mortality (HR=1.013, p=0.003). In the model with only the scores included (4B), GRACE score (HR=1.14, p<0.001) and EuroSCORE II (HR 1.070, p=0.011) were significantly associated with mortality in the entire population, and only GRACE score showed a significant association with long-term mortality (HR=1.013, p=0.005) when excluding in-hospital deaths.

## Discussion

In a real-world population with ACS submitted to CABG during the index hospitalization, among the analyzed ischemic, bleeding, and surgical scores, the best performance was obtained by the GRACE risk score.

Previously, a European study with around 2500 patients with ACS undergoing CABG during the index hospitalization^3^demonstrated an in-hospital mortality rate of 8.1%, similar to the 8.3% obtained in the present publication. From the predictors of in-hospital mortality, our population was four years younger (mean age 67.8 vs 63.5 years, reported OR for age = 1.03) but had a higher proportion of women (reported OR = 1.73, incidences 29.8% vs 22.4%) and previous myocardial infarction (reported OR = 1.83, incidences 31.6% vs 27.1%). Additionally, our population had a higher prevalence of diabetes (39.6% vs 32.1%) and previous cardiac surgery (5.7% vs. 2.2%), with similar left ventricular ejection fraction (50.3% vs 50.7%) and incidence of STEMI (22.8% vs 23%).

**Scores and in-hospital mortality:** Risk scores help identify high-risk patients who may benefit from early invasive strategies or intensified medical therapy, guide the use of antithrombotic agents with bleeding risk consideration, and aid in shared decision-making with patients and families. Previous publications found a positive association of the analyzed scores with short-term mortality.^15 , 17 , 23 - 25^In the present study, all scores showed a significant association with in-hospital mortality when analyzed as continuous variables. When analyzed as categorical variables, the highest odds ratio was obtained with the GRACE score (OR = 8.52 for the entire population, OR = 9.49 for the subset with complete score data). Moreover, the GRACE score was the only score associated significantly and independently with in-hospital mortality in all multivariable models.

Regarding the predictive value of the different scores, analyzed by ROC curves, prior studies of the GRACE risk score have consistently demonstrated AUC measures greater than 0.80.^13 , 22^For the A-H bleeding score, a 0.75 value was described;^25^for TIMI-STEMI, the result from the original publication was 0.78; for TIMI-NSTEACS, 0.65 for all-cause mortality, MI, or urgent revascularization was found,^16^and 0.60 in a subsequent publication considering 30-day mortality.^26^Finally, for the EuroSCORE II, an AUC of 0.81 was reported, and among a broad population undergoing major cardiac surgery (47% isolated CABG).^17^In general, the results from the present study are in line with these publications, with AUC of 0.82, 0.67, 0.78, 0.58, and 0.75, respectively, for GRACE score, A-H bleeding score, TIMI-STEMI score, TIMI-NSTEACS, and EuroSCORE II.

Previous publications compared different risk scores in patients with or suspected of having ACS.^23 , 26 - 30^However, to the best of our knowledge, none has compared risk scores specifically in patients with ACS undergoing CABG during the index hospitalization, much less considering traditional ACS scores along with a bleeding score and a major cardiac surgery risk score. ROC curve analyses showed the highest AUC for the GRACE score; moreover, the GRACE score was the only one associated significantly and independently with in-hospital mortality in all the adjusted models that were developed.

**Scores and long-term mortality:** The role of the various scores in medium/long-term mortality after an episode of ACS has been described previously,^20 , 23 , 27^showing in general good overall performance. As with in-hospital mortality, to the best of our knowledge, this is the first study to compare different risk scores for long-term mortality in ACS patients undergoing CABG during the same hospitalization. In the present study, Kaplan-Meier curves showed the GRACE score with the best performance among the analyzed scores, and the only score associated significantly and independently with long-term mortality in all the adjusted models.

### Limitations

Like all observational studies, ours has several limitations. First, like in any study derived from a retrospective databank, there is a possibility that unaccounted confounders in the adjusted models could have influenced the results. Nonetheless, comparing the ROC curves provides a useful and more comprehensive assessment of the hypothesis. Second, we did not have the data for all the analyzed scores prospectively collected, and there were many with missing data; however, the comparison between the complete dataset and the dataset with all scores available did not show significant differences. Third, our results were derived from a single-center dataset, and the results might not be replicable in other institutions. Fourth, our long-term results must be taken carefully, given our loss to follow-up rates, which may introduce bias and reduce statistical power to find some associations. Fifth, changes in the routine management, including improvements in medical treatment and in PCI and CABG techniques during the period, as well as selection criteria for coronary revascularization strategies, may have influenced our results. Importantly, our institutional protocols were updated constantly according to national and international guidelines. Finally, we were not able to retrieve the STS score, and it was not analyzed in the present study; however, it is important to note that the comparison between STS and EuroSCORE II in Brazilian and Indian populations showed similar results.^31 , 32^

## Conclusion

In patients with ACS who underwent CABG during the index hospitalization, the GRACE score was the only risk score that remained independently associated with both in-hospital and long-term mortality across all developed models, after adjustment for potential confounders. Moreover, the GRACE score showed the best performance in predicting in-hospital deaths. These findings may influence the clinical decision-making process in this high-risk population.

## Supplemental Materials

SUPPLEMENTAL MATERIAL
